# The combination of accent method and phonemic contrast: an innovative strategy to improve speech production on post-stroke dysarthria

**DOI:** 10.3389/fnhum.2023.1298974

**Published:** 2024-01-08

**Authors:** Shengnan Ge, Qin Wan, Yongli Wang, Minmin Yin, Zhaoming Huang

**Affiliations:** ^1^School of Life Sciences, East China Normal University, Shanghai, China; ^2^Department of Rehabilitation Sciences, Faculty of Education, East China Normal University, Shanghai, China; ^3^Department of Special Education, Faculty of Education, Hangzhou Normal University, Hangzhou, China

**Keywords:** speech production, segments, suprasegments, phonemic contrast, accent method, dysarthria with post-stroke

## Abstract

**Introduction:**

Speech production includes segmental and suprasegmental features, which interact and cooperate with each other. Impaired speech production is common in individuals with post-stroke dysarthria. The commonly used phonemic contrast therapy and Accent Method in clinical practice can improve the segmental and suprasegmental aspects, respectively. This study aimed to explore the feasibility and immediate effectiveness of the combination of specific rhythm patterns of the Accent Method and phonemic contrast on speech production.

**Methods:**

Fifteen poststroke dysarthria patients (12 males and three females) first received accentuation task of three rhythm patterns (Largo-slow, Andante-medium, Allegro-fast) and later received speech task in appropriate rhythm patterns combined with phonemic contrast materials and non-phonemic contrast materials. Speech parameters were analyzed by considering speech clarity and prosody.

**Results:**

The results showed that the number of correct target syllables, sentence clarity, and standard deviation of intensity increased significantly, and the average length of pauses and abnormal pause times decreased significantly in Andante (medium) compared to other rhythms. The number of correct target syllables, sentence clarity, and standard deviation of intensity increased significantly compared with those in the non-phonemic contrast in Andante (medium).

**Conclusion:**

The combination of phonemic contrast and Accent Method was verified to have an immediate effect on speech production in Mandarin speakers with post-stroke dysarthria and could be further validated in other diseases with impaired speech production in the clinic in the future.

## 1 Introduction

Impaired speech production in common in dysarthria, which is a common and persistent sequela of stroke and can have a detrimental influence on communication and quality of life, with reported incidence rates as high as 26–44% (Stipancic et al., 2019; De Cock et al., 2020). Post-stroke dysarthria arises from lesions in various locations is largely associated with spastic and unilateral upper motor neuron dysarthria, and to a lesser extent some other subtypes (Dickson et al., 2008). Respiration, phonation, resonance, articulation, and prosody of speech production can be affected in isolation or combination, common in imprecise articulation (involving consonants, less commonly vowels), slow speaking rate, voice disturbances, and reduced prosodic variation (monotony of speech) (Chiaramonte and Vecchio, 2021). These segmental and suprasegmental information of speech production comprise acoustic regularities necessary for improving the processing of neurologically degraded speech signals.

### 1.1 Manifestations of segmental defects and related treatments

Consonant imprecision, irregular articulatory breakdown, and vowel distortion are typical of segmental defects, resulting in reduced speech clarity in specific phonemes (Mackenzie and Lowit, 2007). These manifestations may be due to impaired motor control during speech production (Duffy, 2019). Each phoneme has a specific place and mode of articulation corresponding to it. When dysarthria is impaired in a specific articulatory movement, it first affects the accurate articulation of the phonemes associated with this movement. For instance, when the backward movement of dysarthria’s tongue is limited, it will become difficult to construct the retrolingual phoneme/g/. The patient may construct the pronunciation with the tongue in a relatively front position, which sounds like/d/or even/b/. The patient may also pronounce a distorted phoneme or even omit the pronunciation of retrolingual phoneme directly. Manochiopinig et al. (2008) found that stroke patients are prone to phoneme imprecision, especially in consonant errors. Among the types of consonant errors, substitution was the most common, followed by omission and distortion.

Articulatory motor training, speech clarity training among others (Chiaramonte et al., 2020) are traditional therapies for improving segmental aspect of speech production, including training to increase physiological support for speech via exercises to increase the strength, range, precision, and speed of muscle movements involved in speech production (Duffy, 2019). The ultimate goal of the training is to improve speech intelligibility, that is, to improve the articulation imprecision to allow the listeners to understand the maximum degree. Phonemic contrast therapy is a phonological-based approach to suppress the error patterns of articulatory disturbances (Dodd et al., 2008). Contrast training of minimal phonemic pairs is adopted to increase the articulatory accuracy of confusing phonemes, which differ only in one dimension, making it easier for patients to access fine phonemic details. Mackenzie and Lowit (2007) found significant improvements in articulatory accuracy after minimal phonemic contrast therapy in post-stroke dysarthria.

### 1.2 Manifestations of suprasegmental defects and related treatments

Suprasegmental defects, including prosodic disorders, are commonly characterized by weakened breathing, abnormal pauses and speech speed, monopitch, and monoloudness (Hernandez et al., 2020; Borrie and Lansford, 2021). Mackenzie (2011) searched relevant studies on post-stroke dysarthria features and summarized the suprasegmental characteristics of post-stroke dysarthria in terms of reduction in rate, prolonged intervals or syllables, monotone, reduced inflection, pitch, stress, and loudness variation.

The Accent Method (AM) has been widely applied to improve suprasegmental characteristics through changes in properties, such as fundamental frequency and intensity (Murdoch and Theodoros, 1998; Khidr, 2003; Shiromoto, 2003; Zsiga, 2012; Bassiouny et al., 2019; Othman et al., 2021). AM should be done within one breath using a specific rhythm. Participants are required to read starting with soft voice to establish appropriate vocal cord vibration patterns, and to read syllables with undulating intonation and stress in one breath to improve monopitch and monoloudness. Compared with other prosodic approaches, AM considers the progressive difficulty of the speech materials in training, such as the transition from vowels to connected speech, and focuses on the comprehensive application of rhythm, intonation, and stress (Kotby and Fex, 1998).

The rhythm of AM is divided into three types: Largo (slow), Andante (medium), and Allegro (fast) (Kotby et al., 1991). Largo was the slowest, with one accentuation; Andante was medium, with three accentuations; and Allegro was the fastest, with five accentuations. Strict AM emphasizes the gradual use of three rhythm patterns, namely the transition from Largo (slow) to Andante (medium), and finally to Allegro (fast) (Kotby and Fex, 1998). However, for many individuals with speech production impairment, including post-stroke dysarthria, abnormal speech speed as a typical clinical manifestation (Duffy, 2019) could greatly interfere with speech clarity and prosody during speech production (Dagenais et al., 2006; Knowles et al., 2021). Training or stimulation under an inappropriate rhythm pattern may aggravate speech deficits. Therefore, it is necessary to adjust the common rhythm patterns, such as finding a rhythm suitable for post-stroke dysarthria.

### 1.3 Necessity of combined treatment of segmental and suprasegmental aspects

Speech carries segmental (phonemic) as well as suprasegmental (non-phonemic) detail (Singh et al., 2016), which are both an integral part of the pronunciation system where the production of one can influence the other (Wang, 2022). Neither in English nor in Mandarin, suprasegmental cannot exist independently from segmental information for both single syllable and continuous speech (Zielinski, 2015). English is a stress-timed language, which is characterized by abundant fundamental frequency variation in phonemes and words during speech production. Similar to stress in English, tone is a part of the suprasegmental phonology in Mandarin (Wang et al., 2015). Mandarin is a tonal language. Tones (prosodic elements) must be attached to vowels during speech production of syllables or lexical items. During the speech production, it is necessary not only to consider the attachment of tones to vowels, but also to rely on prosodic cues (rhythm, intonation, and stress) to achieve fluent expression.

In addition, previous studies have demonstrated that the two hemispheres differ in the segmental and suprasegmental characteristics of speech production (Sammler et al., 2010). The left hemisphere is the dominant hemisphere for speech and language processing in most people, and is responsible for processing segmental characteristics (e.g., phonemes and words) (Blank et al., 2002; Flinker et al., 2015). The right hemisphere is the key area for prioritizing and controlling the suprasegmental characteristics, such as prosody and paralanguage (Lindell, 2006; Haider et al., 2019). In normal speech, there is a dynamic interaction between the two cerebral hemispheres (Friederici and Alter, 2004), which builds the brain basis for the coordination and integration of phonemic and prosodic characteristics during speech.

Based on the available evidence, there is no one size fits all strategy to improve speech production comprehensively for individuals with dysarthria (Fletcher et al., 2017). To apply these strategies to patients with specific diseases and treatment goals, a number of approaches need to be adapted for training. Kim and Jo (2013) performed a modified Accent Method that integrated musical elements into accentuated vocalization for mixed dysarthria after stroke, and the results indicated an improvement in speech motor coordination, including respiration, phonation, articulation, resonance, and prosody. However, they only focused on articulation at the monophonic and consonant levels, without considering articulation at the syllable or continuous speech levels, which usually occur more frequently in communication.

The goal of speech therapy is to maximize speech intelligibility and restore the fluent speech, involving the collaboration of connected speech and specific prosodic changes (Chiaramonte et al., 2020). It has been reported that comprehensive treatment is capable of improving speech production from multiple functional aspects (Aten, 1988), such as the combined training of articulation and prosody (Palmer and Enderby, 2007). Therefore, it is necessary to simultaneously attach importance to and consider the segmental and suprasegmental aspects of speech production as a whole to comprehensively improve or recover speech production.

To achieve the therapeutic goal of prosodic and clear connected speech and the purpose of restoring the coordination between the left and right hemispheres for Mandarin speakers with impaired speech production, we wondered whether speech stimulation with a combination of phonemic contrast and AM elements can quickly improve the speech clarity and prosody. The purpose of this study was to explore a suitable rhythm pattern and to explore the feasibility and influence of phonemic contrast combined with specific rhythm patterns on improving speech clarity and prosody of speech production. We selected segmental parameters (number of correct target syllables, sentence clarity) and suprasegmental indicators (average length of pause, abnormal pause times, speech rate, standard deviation of fundamental frequency, and standard deviation of intensity) to observe the speech clarity and prosody of post-stroke dysarthria before and after the task.

## 2 Materials and methods

### 2.1 Participants

A total of fifteen patients (twelve males and three females) with an average age of 64.267 ± 9.903 years were randomly selected from a rehabilitation hospital in Shanghai. All participants had a course of disease of more than 1 month. All participants were diagnosed with stroke by CT, MRI and other imaging examinations, and the damage site of each participant’s brain region was identified. Moreover, the speech status, subtypes and severity of dysarthria were determined according to Frenchay Dysarthria Assessment combined with auditory-perceptual assessment. Each patient needed to be assessed using the “Chinese Articulation Assessment” to judge their articulation status. All participants were from Shanghai, China, and spoke Mandarin. They were classified into middle socioeconomic status based on the degree of education, income, and occupation. None of the participants had hearing or vision impairment. Participants were able to understand the instructions and cooperated to complete the experiment. Patients with moderate-to-severe aphasia (or comprehension aphasia), speech apraxia, or cognitive impairment were excluded from this study. Participants would also be excluded from the study if they developed symptoms such as colds, stuffy nose, or cough within 2 weeks before the study. Basic information on the participants is presented in Table 1. This study included 8 spastic, 4 mixed, and 2 unilateral upper motor neuron dysarthria.

**TABLE 1:** Demographic information, diagnosis, lesion sites, classification, and severity of the participants.

	Sex	Age	Diagnosis	Lesion sites	Classification	Severity
N1	M	81	Cerebral infarction	BG; BS; FL	Mixed (spastic-hypokinetic)	Severe
N2	M	49	Cerebral infarction	BG; BS	Spastic	Severe
N3	M	66	Cerebral hemorrhage	BG	UUMN	Mild
N4	M	66	Cerebral infarction	BG; CEO; THA	Mixed (spastic-ataxic)	Mild
N5	M	65	Cerebral hemorrhage	THA; LV; BG; THA	Spastic	Mild
N6	M	63	Cerebral infarction	BG; CS	Spastic	Moderate
N7	M	74	Cerebral infarction	LV; BG	Spastic	Mild
N8	F	58	Cerebral infarction	BG; CEO; BS	Mixed (spastic-ataxic)	Severe
N9	M	43	Cerebral hemorrhage	BG	UUMN	Mild
N10	M	64	Cerebral infarction	CC; CS; BG; BS	Spastic	Moderate
N11	M	67	Cerebral infarction	LV; FL	Spastic	Severe
N12	F	54	Cerebral infarction	BG; FL; TL	Mixed (spastic-flaccid)	Moderate
N13	M	70	Cerebral infarction	BG; FL; CEO; BS; THA	Mixed (spastic-ataxic)	Severe
N14	F	72	Cerebral infarction	FL; TL	Spastic	Moderate
N15	M	72	Cerebral infarction	BS; BG	Spastic	Moderate

N, number; M, male; F, female; BG, basal ganglia; CS, centrum semiovale; FL, frontal lobe; BS, brain stem; CEO, cerebellum; THA, thalamus; LV, lateral ventricle; CC, corpus callosum; TL, temporal lobe; UUMN, unilateral upper motor neuron.

This study was approved by the University of Human Research Ethics Committee. Appropriate informed consent and details were provided to all participants before the study.

### 2.2 Experiment 1

#### 2.2.1 Speech materials

According to the principles of generality and practicability, the first experiment compiled three seven-word-length sentences with equal difficulty and simple structure composed of high-frequency words to explore the appropriate rhythm pattern for post-stroke dysarthria, including “shāng chǎng lǐ yǒu diàn yǐng yuàn,” “xué xiào lǐ yǒu tú shū guǎn,” and “kè tīng lǐ yǒu diàn shì jī” in Mandarin (i.e., “There is a cinema in the shopping mall,” “There is a library in the school,” and “There is a television in the living room” in English, respectively). In the sentences, “diàn yǐng yuàn (cinema),” “tú shū guǎn (library),” and “diàn shì jī (television)” were used as the target syllables to be accentuated.

#### 2.2.2 Procedure

**FIGURE 1:**
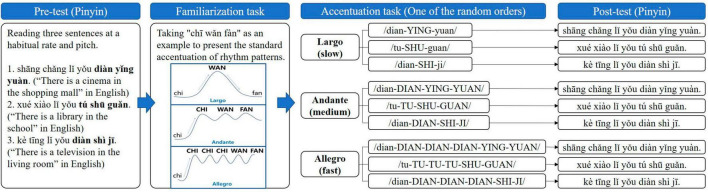
The procedure of Experiment 1, including pre-test, familiarization task, accentuation tasks, and post-test. Bold, target stimuli; lower case, reading starting with the soft voice; uppercase, reading with accentuated voice (increased pitch, stress, and loudness); hyphens, reading in one breath.

The process of Experiment 1 is presented in Figure 1, including the following steps. (1) Pre-test. Participants were asked to read and record three sentences in a quiet environment at a habitual speaking rate and pitch. (2) Familiarization task. Since none of the participants had been received the training of the Accent Method before this study, we used the simple word “chī wǎn fàn (eat dinner in English)” as the example to familiarize participants with the Accent Method before the experimental stimulus. The example sentence “wǒ yào chī wǎn fàn” in Mandarin (i.e., “I’m going to eat dinner” in English) in which “chī wǎn fàn (eat dinner)” was used as the target syllables to present and explain the standard accentuated play of three rhythm patterns. Taking the Largo (slow)/chi-WAN-fan/of the target syllables “chī wǎn fàn” as the example, lowercase meant to be read starting with the soft voice, uppercase meant to be read with accentuated voice (increased pitch, stress, and loudness), and hyphens meant it should be read in one breath. Uppercase, lowercase, and hyphens in Andante (medium)/chi-CHI-WAN-FAN/and Allegro (fast)/chi-CHI-CHI-CHI-WAN-FAN/represented the same meaning as Largo (slow). (3) Accentuation tasks and post-test. Three rhythm patterns were used to perform the accentuation task for the three target syllables. Since we needed to have all participants complete the accentuation stimulus of the three target words in all three rhythmic patterns, participants may have practice effect or fatigue reaction. In order to avoid these problems, we used a computer program to generate a total of nine random orders in which participants received accentuation stimuli in one of the random orders. After each rhythm-type stimulus, participants rested for 2 h before receiving the next rhythm-type stimulus. For example, the accentuation task in Figure 1 presents one of these random stimulus orders. The specific stimulation process was as follows: the experimenter played standard audio of the accentuation, and explained the essentials of the action about the accentuation to guide participants to imitate. At the beginning of the task, the experimenter used gestures to prompt participants, and then gradually revoked the gestures. After performing the specific accentuation task for each target syllables, the participants were asked to read the sentence containing these target syllables at the habitual speaking rate and pitch as post-test. Then, the participants received the next accentuation task with randomly presented target syllables and rhythm pattern.

#### 2.2.3 Data collection

We used a digital recorder (Sound Forge 9.0) with a 44.1-kHz sampling rate and 16-bit quantization rate to collect the speech samples read by the participants. During the recording, the microphone was placed at a 45-degree angle to the desktop and the participant’s mouth, and the microphone was 8–12 cm away from the participant’s mouth. The entire recording process was completed under 40 dB SPL ambient noise.

#### 2.2.4 Data analysis and statistical analysis

The number of correct target syllables, sentence clarity, average length of pause (Barnett et al., 2020), abnormal pause times, speech rate (Sevitz et al., 2021), standard deviation of fundamental frequency (F0SD) (Chiaramonte and Vecchio, 2021), and standard deviation of intensity (intensity SD) (Behrman et al., 2020) were selected as the indicators to analyze the speech status. The target syllables and sentences to be judged in the two experiments were inconsistent. In the first experiment, three target sentences were used (“shāng chǎng lǐ yǒu diàn yǐng yuàn,” “xué xiào lǐ yǒu tú shū guǎn,” “kè tīng lǐ yǒu diàn shì jī” in Pinyin, respectively), and the bold ones were the target syllables. The number of correct target syllables was the number of clear target syllables in the corresponding sentence, and sentence clarity was the average proportion of clear syllables in the target sentences. Two trained speech therapists judged the number of correct target syllables and sentence clarity. Inter-rater agreement between the two raters exceeded 0.9. In this study, a speech disorder measuring instrument, Dr. Speech, was used for the data analysis (Kim et al., 2019). Abnormal pause is a silent segment with a duration of ≥300 ms during speech (Raupach, 2011; Skodda, 2011), and prosodic cues for rhythm, intonation, and stress are primarily related to voice variations in duration, intensity, and fundamental frequency (Grant and Walden, 1996). F0SD and intensity SD are the dispersions of the speech fundamental frequency and intensity, respectively. The calculation formulas for the remaining indicators are as follows:


$$\begin{array}{c} \mathrm{Sentence}\mathrm{clarity}=\mathrm{the}\mathrm{number}\mathrm{of}\mathrm{clear}\mathrm{syllables}/\\ \mathrm{total}\,\mathrm{number}\,\mathrm{of}\,\mathrm{syllables}*100\% \end{array}$$

$$\begin{array}{c} \mathrm{Average}\mathrm{length}\mathrm{of}\mathrm{pause}=\mathrm{total}\mathrm{pause}\mathrm{duration}/\\ \mathrm{number}\,\mathrm{of}\,\mathrm{pauses} \end{array}$$

Speech⁢rate=number⁢of⁢syllables/total⁢duration⁢of⁢sentence

Single-factor repeated measures experimental design was adopted, with rhythm patterns (Largo, Andante, and Allegro) as the independent variable, and speech clarity and speech prosody features as the dependent variables. SPSS (version 23.0) was used for statistical analyses. The statistical results of Sphericity Assumed were referred if the sphericity assumption was satisfied, otherwise the statistical results of Greenhouse-Geisser were referred. Statistical results were considered significantly different when the *p*-value was < 0.05.

### 2.3 Experiment 2

The first experiment reported that Andante (medium) was the appropriate rhythm pattern, and it was then combined with phonemic contrast and non-phonemic contrast materials to explore the suitable combination to further improve the speech clarity and prosody of speech production.

#### 2.3.1 Speech materials

The materials we selected were Mandarin sentences that contained minimal consonant phoneme pairs. There are 21 consonants in Mandarin. The combinations of these consonant phonemes and specific vowels form the standard pronunciations in Mandarin Pinyin, such as/po/,/te/,/bo/,/fo/,/de/,/ge/,/ji/,/qi/,/le/,/ri/. These 21 consonant phonemes can be divided into pairs to form 25 minimal consonant phoneme pairs according to the phoneme position, mode, and inspiratory characteristics. We compiled a speech material library consisting of 25 sentences containing minimal consonant phoneme pairs. each sentence was eight-word-length and contained two high-frequency disyllabic words consisting of the target minimal phoneme pairs. The disyllabic words were in the same position in the sentence (middle and end of the sentence). Before the experiment, all participants were given the “Chinese Articulation Assessment” to judge the impaired phoneme pairs (Alsaad et al., 2019). We found that they were impaired in the following phonemic pairs, including/p-t, b-f, d-g, b-p, j-q, l-r/. Therefore, sentences containing the above six phonemic pairs were selected from the speech material library as target sentences in Experiment II. These sentences included:/p-t/—“wǒ yòng **pú tao** zhì zuò **tián pǐn** (‘I make desserts from grapes.’ in English),”/b-f/—“tā zài **fā bù** jié juě **bàn fǎ** (‘He is publishing a solution.’ in English),”/d-g/—“wǒ qù **guǎng dōng** yīng pìn **dī gē** (‘I went to Guangdong to apply for a taxi driver.’ in English),”/b-p/—“tā jiāng **bǔ pǐn** zhuāng jìn **pí bāo** (‘He put the tonic in his bag.’ in English),”/j-q/—“wǒ zài **qīng jié** kāng fù **jī qì** (‘I’m cleaning the rehabilitation machine.’ in English),”/l-r/—“tā hé **lǎo rén** qù mǎi **rǔ lào** (‘He went to buy cheese with the old man.’ in English).” The bolded disyllabic word in each sentence was the target stimulus.

#### 2.3.2 Procedure

**FIGURE 2:**
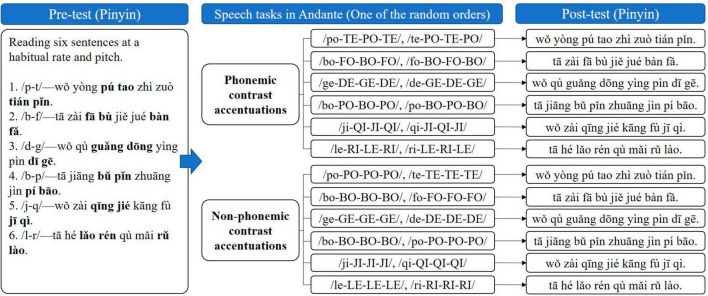
The procedure of Experiment 2, including pre-test, speech tasks in Andante, and post-test. Bold, target stimuli; lower case, reading starting with the soft voice; uppercase, reading with accentuated voice (increased pitch, stress, and loudness); hyphens, reading in one breath.

The process of Experiment 2 is presented in Figure 2, including the following steps. (1) Pre-test. Participants were asked to record and read six sentences in a quiet environment at the habitual speaking rate and pitch as the pre-test. (2) Speech tasks in Andante (medium) and post-test. In Mandarin, the combination of consonant and specific monophthong forms the standard pronunciation, such as/po/,/te/,/bo/,/fo/,/de/,/ge/,/ji/,/qi/,/le/,/ri/. Based on the results of Experiment 1 that Andante (medium) was the appropriate rhythm type, Experiment 2 combined Andante (medium) with the phonemic contrast and non-phonemic contrast forms of the above standard pronunciation to perform the speech accentuation tasks. In the phonemic contrast combined accentuation task, the standard pronunciations of two consonants that can be combined into the minimal phonemic pair are alternated in one accentuation, such as/bo-PO-BO-PO/and/po-BO-PO-BO/. In the non-phonemic contrast combined accentuation task, the standard pronunciations of these two consonants was repeated in two accentuations, respectively, such as/bo-BO-BO-BO/and/po-PO-PO-PO/. All accentuation materials and corresponding visual cues for Andante (medium) were presented to participants in a randomized order in PowerPoint. The experimenter played standard audio, explained the essentials of the accentuation, and used gestures to guide the participant to imitate, then gradually revoked the prompts for the gestures. After performing the specific speech task in Andante (medium) for each target syllables, the participants were asked to read the sentence containing these target syllables at the habitual speaking rate and pitch as post-test. Participants then received the next randomly presented task.

#### 2.3.3 Data analysis and statistical analysis

The number of correct target syllables, sentence clarity, average length of pause, abnormal pause times, speech rate, standard deviation of fundamental frequency (F0SD), and standard deviation of intensity (intensity SD) were selected as the indicators to analyze the speech status. Six target sentences with phonemic contrast words were used (/p-t/—“wǒ yòng pú tao zhì zuò tián pǐn,”/b-f/—“tā zài fā bù jiě jué bàn fǎ,”/d-g/—“wǒ qù guǎng dōng yīng pìn dī gē,”/b-p/—“tā jiāng bǔ pǐn zhuāng jìn pí bāo,”/j-q/—“wǒ zài qīng jié kāng fù jī qì,”/l-r/—“tā hé lǎo rén qù mǎi ru lào”), and the ones in bold were the target syllables.

This experiment adopted a single-factor repeated measures experimental design, with speech corpus (phonemic contrast or non-phonemic contrast) as the independent variable and above indicators as the dependent variables. SPSS (version 23.0) was used for statistical analyses. The statistical results of Sphericity Assumed were obtained if the sphericity assumption was satisfied, otherwise the statistical results of Greenhouse-Geisser were obtained. Statistical results were considered significantly different when the *p*-value was < 0.05.

## 3 Results

### 3.1 Experiment 1

The descriptive and significant results of the indicators before and after the stimuli of the three AM rhythm patterns in the first experiment are shown in Table 2. The main effects of rhythm patterns were significant in the number of correct target syllables, sentence clarity, average length of pause, abnormal pause times, and intensity SD (*F* = 19.525, *p* < 0.001; *F* = 27.633, *p* < 0.001; *F* = 4.591, *p* = 0.021; *F* = 6.720, *p* = 0.004; *F* = 7.970, *p* < 0.001), but not in speech rate and F0SD (*F* = 0.168, *p* = 0.917; *F* = 0.324, *p* = 0.808), demonstrating a significant difference in speech clarity, pause, and loudness of post-stroke dysarthria before and after the repetition of accentuation tasks.

**TABLE 2:** Descriptive statistics and analysis of variance results of parameters on segmental and suprasegmental aspects before the task (pre-test) and after the tasks of different rhythm patterns (largo, andante, and allegro).

		Number of correct target syllables	Sentence clarity	Average length of pause	Abnormal pause times	Speech rate	F0SD	Intensity SD
Pre-test		1.711 ± 0.469	0.579 ± 0.164	0.274 ± 0.224	0.467 ± 0.451	2.701 ± 0.783	22.667 ± 11.990	8.952 ± 1.343
Post-test	Largo	1.956 ± 0.533	0.657 ± 0.202	0.190 ± 0.170	0.200 ± 0.303	2.668 ± 0.679	21.222 ± 11.406	9.559 ± 1.786
	Andante	2.444 ± 0.638	0.778 ± 0.196	0.151 ± 0.143	0.067 ± 0.187	2.706 ± 0.574	21.844 ± 10.791	11.405 ± 3.271
	Allegro	2.133 ± 0.602	0.689 ± 0.176	0.183 ± 0.128	0.156 ± 0.248	2.762 ± 0.640	21.200 ± 12.207	9.837 ± 2.153
*df*		2.163	3	1.869	1.982	3	3	3
F		19.525***	27.633***	4.591*	6.720**	0.168	0.324	7.970***
*p*		<0.001	<0.001	0.021	0.004	0.917	0.808	<0.001

F0SD, standard deviation of fundamental frequency; Intensity SD, standard deviation of intensity.

*p < 0.05;

**p < 0.01;

***p < 0.001.

**FIGURE 3:**
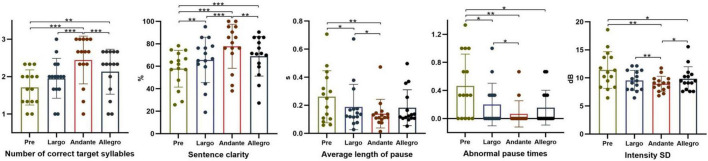
Effects of different rhythm patterns on the parameters on segmental and suprasegmental aspects. *p < 0.05; **p < 0.01; ***p < 0.001.

The results of the multiple comparisons of the indicators are shown in Figure 3. The number of correct target syllables, sentence clarity, and intensity SD in Andante (medium) were significantly increased compared with the pre-test, Largo (slow), and Allegro (fast) (*p* < 0.001, *p* < 0.001, *p* = 0.002; *p* < 0.001, *p* < 0.001, *p* = 0.007; *p* < 0.001, *p* = 0.001, *p* = 0.033); the average length of pause and the abnormal pause times were significantly decreased compared with the pre-test and Largo (slow) (*p* = 0.009, *p* = 0.002; *p* = 0.017, *p* = 0.028). It was indicated that the Andante (medium) rhythm patterns can improve speech clarity and prosody more obviously.

### 3.2 Experiment 2

Descriptive and significant results for the indicators before and after receiving the stimulus of post-stroke dysarthria are shown in Table 3. From the descriptive statistical results, it was found that the indicators after receiving any stimulus had improved to a certain extent compared with those before stimulation. The main effects of the training corpus were significant in the number of correct target syllables, sentence clarity, abnormal pause times, speech rate, F0SD, and intensity SD (*F* = 41.044, *p* < 0.001; *F* = 78.395, *p* < 0.001; *F* = 8.477, *p* = 0.001; *F* = 6.883, *p* = 0.004; *F* = 4.418, *p* = 0.022; *F* = 6.771, *p* = 0.010), but not in the average length of pause (*F* = 0.742, *p* = 0.437).

**TABLE 3:** Descriptive statistics and analysis of variance results of parameters on segmental and suprasegmental aspects before the task (pre-test) and after the tasks of combining Andante and speech materials (phonemic contrast and non-phonemic contrast).

		Number of correct target syllables	Sentence clarity	Average length of pause	Abnormal pause times	Speech rate	F0SD	Intensity SD
Pre-test		2.589 ± 0.667	0.708 ± 0.162	0.386 ± 0.332	1.067 ± 0.626	2.293 ± 0.535	20.422 ± 6.502	11.962 ± 2.116
Post-test	Phonemic contrast	3.356 ± 0.475	0.858 ± 0.123	0.329 ± 0.340	0.622 ± 0.536	2.638 ± 0.564	23.444 ± 6.044	13.461 ± 2.000
	Non-phonemic contrast	2.867 ± 0.513	0.768 ± 0.137	0.367 ± 0.409	0.700 ± 0.578	2.601 ± 0.668	22.222 ± 7.144	12.050 ± 2.241
*df*		2	2	1.323	2	2	2	1.458
F		41.044***	78.395***	0.742	8.477**	6.883**	4.418*	6.771*
*P*		<0.001	<0.001	0.437	0.001	0.004	0.022	0.01

F0SD, standard deviation of fundamental frequency; Intensity SD, standard deviation of intensity.

*p < 0.05;

**p < 0.01;

***p < 0.001.

**FIGURE 4:**
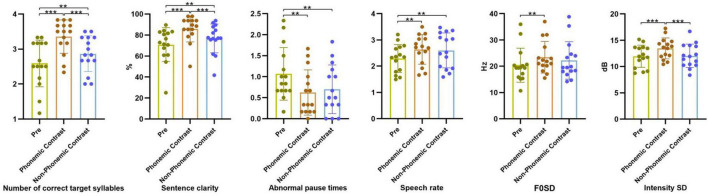
Effects of different speech materials in Andante on the parameters on segmental and suprasegmental aspects. **p < 0.01; ***p < 0.001.

Figure 4 shows the results of the multiple comparisons of the indicators of the training corpus. The results demonstrated that the number of correct target syllables, sentence clarity, speech rate, F0SD, and intensity SD under the phonemic contrast stimulus were significantly higher than those in the pre-test (*p* < 0.001, *p* < 0.001, *p* = 0.005, *p* = 0.008, *p* = 0.008, respectively), and abnormal pause times was significantly decreased (*p* = 0.005). The number of correct target syllables, sentence clarity, and intensity SD under the phonemic contrast stimulus were significantly higher than under the non-phonemic contrast stimulus (*p* = 0.001, *p* = 0.001, *p* = 0.022). The number of correct target syllables, sentence clarity, and speech rate under the non-phonemic contrast stimulus significantly increased (*p* = 0.001, *p* = 0.001, *p* = 0.009), and abnormal pause times significantly decreased (*p* = 0.005) compared with the pre-test. Therefore, the combined stimuli of phonemic-contrast and the Andante (medium) of AM can improve speech clarity and prosody.

## 4 Discussion

This study aimed to explore a suitable rhythm pattern and the feasibility and influence of the combination of specific rhythm patterns and phonemic contrasts on improving speech clarity and prosody of speech production. The results indicated that speech production improved significantly after the stimulus of the Andante (medium) rhythm combined with phonemic contrast materials. The combination of phonemic contrast and Accent Method was integrated with segmental and suprasegmental aspects to improve speech production in terms of clarity, rhythm, intonation, and stress.

### 4.1 Appropriate accentuated rhythm pattern for improving speech production

In terms of prosody, the results of the first experiment showed that the average length of pause and abnormal pause times were significantly reduced and intensity SD was significantly increased after the Andante (medium) stimuli. An appropriate accentuated rhythm pattern helps to develop optimal breath support, together with controlling the duration, pause, speed, and loudness (Khidr, 2003). The speech speed of post-stroke dysarthria is usually slow, so the slow rhythm (Largo) may further lead to delayed speech due to the limited number of syllables in one breath due to limited expiratory support. For Allegro (fast), dysarthria usually compensates by reducing the range of articulatory movements or coordination between articulatory organs in order to produce enough syllables in one breath at a fast rhythm, which would also have adverse effects in the long term. Lastly, medium tempo (Andante) can give dysarthria enough time to move to the target articulation position so the speech movement can be stable and accurate for clear speech, which is verified in this study. In addition, it was reported that Andante of AM causes greater flexibility in controlling suprasegmental features (Shiromoto, 2003), which was consistent with the results of the present study, namely that the control of laryngeal and respiratory muscles, as well as the coordination of these muscles were improved in dysarthria after Andante (medium) stimuli.

Changes in the suprasegmental aspect had an impact on the segmental aspect. It has been reported that variability in acoustic cues such as intensity and pitch could improve segmentation (Höhle et al., 2020). This validated the articulation-related results of the first experiment; the number of correct target syllables was significantly increased after the Andante (medium) stimulus of AM compared with the pre-test and the other two rhythm patterns. The reason for this result was that the speed of Andante (medium) was more consistent with the speed of daily communication (Kotby and Fex, 1998), which can ensure the enough time to move to the target articulatory position at this speed so that the speech movement can be more stable and accurate to better speech. Too fast or too slow would lead to unclear speech and would be bad for the improvement of speech intelligibility. A faster rhythm would cause patients to compensate by reducing the range of articulation movement or the coordination between articulation motor organs to articulate adequate syllables in one breath. Although reduced speech rate has been promoted as a cueing strategy for improving speech intelligibility in dysarthria (Van Nuffelen et al., 2010; McAuliffe et al., 2014), little consistent evidence has appeared on the effect of reduced speech rate on speech intelligibility outcomes (Fletcher et al., 2017), and not all patients with dysarthria have obtained intelligibility benefits from procedures using these cueing strategies (Cannito et al., 2012). This finding is consistent with the results of the present study. The slower rhythm would cause dysarthria to appear procrastinated during speech production, and the number of words produced per breath was reduced because of the limitation of expiratory support, and speech intelligibility was reduced.

Interestingly, many participants in this study presented with basal ganglia damage. The basal ganglia was considered to be a potentially important structure for recognizing and interpreting prosody, and it constituted the core structure of the functional network of prosody together with the prefrontal and parietal cortex (Rymarczyk and Grabowska, 2007; Wildgruber et al., 2009). It was reported that the basal ganglia, which provided a key mechanism for reinforcing the behavioral significance of prosodic patterns and other temporal representations derived from cue sequences, played a crucial role in intonation and prosodic processing (Pell and Leonard, 2003). Prosodic variation is primarily a global pitch alteration over an extended time window. One of the functional advantages afforded by the basal ganglia during information processing is to enable organisms to adapt to changing temporal contexts (Lieberman, 2000), which was consistent with the results of this study, the best stimulation effects were achieved by coordinating the changes of intonation and stress in Accent Method through the appropriate tempo.

### 4.2 Andante rhythm combined with the phonemic contrast for improving speech production

Consonants are in word-initial position and account for the first few milliseconds of the syllables in Mandarin, which are easily ignored. When the speech task is more challenging (e.g., continuous speech), the assignment of consonants decreases. Post-stroke dysarthria is prone to consonant substitution errors in consonant error categories (Manochiopinig et al., 2008). These easy-to-substitute consonants usually differ only in one characteristic. They were combined into pairs of minimal phonemic contrasts, which were used in AM to significantly improve speech clarity in words and sentences, reflecting a significant increase in the number of correct target syllables and sentence clarity in the second experiment. Although the non-phonemic contrast corpus could also improve the clarity of dysarthria, it only considered the improvement of a single phoneme and did not consider substitution errors.

In addition, suprasegmental aspects may change as segmental aspects change (Patel, 2002), with boundaries of the two often crossing and coinciding (Xu, 2004; Friederici et al., 2007). Clear and fluent speech is ensured by the interaction and coordination of speech processing streams via the corpus callosum between segmental characteristics dominated by the left hemisphere and suprasegmental characteristics dominated by the right hemisphere (Sammler et al., 2010). If one of them or the coordination between the two is impaired, it would lead to difficulties in speech production and reduced speech intelligibility. Treatments that combine segmental and suprasegmental aspects, according to the neural mechanisms of the brain, can comprehensively improve speech production in patients.

Prosodic changes (e.g., stress, pitch, and intensity) could attract patients’ attention to phonemic training and obtain a good curative effect (Höhle et al., 2020), which was similar to the results of this study. Comparing the stimulus combining Andante (medium) and two kinds of speech materials (phonemic contrast and non-phonemic contrast) in the second experiment, abnormal pause times of post-stroke dysarthria was significantly reduced, and speech rate, F0SD, and intensity SD were increased significantly under the stimulus of phonemic contrast combining with Andante (medium), indicating that articulatory and prosodic disturbances were alleviated to improve the speech production of post-stroke dysarthria by the stimulus of Andante (medium) rhythm combined with phonemic contrast. In addition, it was easier to access delicate phonetic detail in minimal phonemic pairs when the changes in specific prosodic cues were salient (such as adding significant pitch and intensity changes in phonemic contrast), which confirmed the strong connection between AM and phonemic contrast (Archer et al., 2014).

Tonal languages are one of the major categories of world’s languages. Although the characteristics of Mandarin varied other languages, the combination of segmental and suprasegmental characteristics, which was the basis of this study, was not only found in studies of Mandarin, but also found in studies of many other languages (Zielinski, 2015; Singh et al., 2016). Mandarin is a tonal language, and tones are only part of the suprasegments. The common suprasegmental characteristics of Mandarin and other languages include intonation, rhythm, stress, among others, which are all attached to the segments (Wang et al., 2015). In terms of segments, although there are special syllabic structures in Mandarin, these syllables are essentially composed of consonants and vowels (Wang, 2022). In this study, we considered the combination of segmental and suprasegmental features for training. Therefore, for patients with speech production impairments speaking languages other than Mandarin, we can also select segmental phonemes that meet the characteristics of the language and accentuation stimuli to complete the training, so as to achieve the goal of combining segments with suprasegments. In addition to Mandarin speakers, we expect this approach will be effective in individuals with speech production impairment speaking other languages.

### 4.3 Limitations

This study is a pilot study, the low sample size was selected to the study and preliminarily verify the effectiveness of this innovative method. In the future, we will expand the sample size and standardize the training according to the treatment strategy, so as to obtain positive training effects. Additionally, specifics about the dysarthria types, speech severity, duration of disease course, and cognitive state could be considered in future studies for clinical applications.

## 5 Conclusion

In summary, this study focused on the feasibility and immediate effectiveness of a combination of specific rhythm patterns and phonemic contrast for improving speech production in post-stroke dysarthria in Mandarin. The results showed that the combination of Andante (medium) rhythm and phonemic contrast was salient in improving speech clarity and prosody, and may become an effective method for improving speech production. The key points of the strategy are as follows: (1) Using Andante (medium) as the accentuated rhythm. (2) Using phonemic contrast syllables as training materials. In the future, we will further verify the applicability of this strategy in other speech production deficit populations.

## Data availability statement

The raw data supporting the conclusions of this article will be made available by the authors, without undue reservation.

## Ethics statement

The studies involving humans were approved by the East China Normal University of Human Research Ethics Committee. The studies were conducted in accordance with the local legislation and institutional requirements. The participants provided their written informed consent to participate in this study. Written informed consent was obtained from the individual(s) for the publication of any potentially identifiable images or data included in this article.

## Author contributions

SG: Data curation, Formal analysis, Investigation, Methodology, Visualization, Writing – original draft, Writing – review and editing. QW: Data curation, Formal analysis, Methodology, Supervision, Visualization, Writing – review and editing. YW: Data curation, Formal analysis, Investigation, Methodology, Visualization, Writing – review and editing. MY: Data curation, Formal analysis, Investigation, Visualization, Writing – review and editing. ZH: Funding acquisition, Methodology, Supervision, Visualization, Writing – review and editing.
